# The Association Between the Hemoglobin Glycation Index and Cardiometabolic Diseases: A Mini‐Review

**DOI:** 10.1111/jch.70092

**Published:** 2025-07-15

**Authors:** Qing‐Yun Wu, Li‐Rong Mo, Jing Nan, Wan‐Zhong Huang, Qiang Wu, Qiang Su

**Affiliations:** ^1^ Department of Cardiology Jiangbin Hospital of Guangxi Zhuang Autonomous Region Nanning Guangxi China; ^2^ Department of Cardiology Affiliated Hospital of Guilin Medical University Guilin Guangxi China; ^3^ Department of Cardiology and Macrovascular Disease Beijing Tiantan Hospital Capital Medical University Beijing China; ^4^ Senior Department of Cardiology the Sixth Medical Center Chinese PLA General Hospital Beijing China

**Keywords:** cardiovascular diseases, glycated hemoglobin, hemoglobin glycation index, insulin resistance, metabolic disorders

## Abstract

The hemoglobin glycation index (HGI) has emerged as a pivotal biomarker for evaluating long‐term glycemic control, offering a more comprehensive assessment compared with conventional glycated hemoglobin (HbA1c) measurements. Elevated HGI levels are significantly correlated with the incidence of cardiometabolic diseases (CMDs). This review synthesizes current evidence on the clinical utility of the HGI across coronary artery disease (CAD), hypertension, heart failure (HF), diabetes mellitus (DM), serum uric acid (SUA) levels, and nonalcoholic fatty liver disease (NAFLD), thereby providing clinicians with an enhanced framework for precise disease stratification, therapeutic optimization, and prognostic prediction.

## 1 Introduction

The increasing global prevalence of cardiometabolic diseases (CMDs) underscores the urgent need for effective risk assessment and management strategies [1]. The hemoglobin glycation index (HGI), which is an emerging metric for evaluating interindividual variability in glycated hemoglobin (HbA1c), has garnered increasing attention in the scientific community [2]. By quantifying differences in HbA1c levels among individuals, the HGI provides novel insights into glycemic stability, thereby offering critical perspectives for understanding the pathogenesis of CMDs.

CMDs, which include hypertension, diabetes mellitus (DM), coronary artery disease (CAD), and related conditions, represent a major public health challenge worldwide [3]. These diseases not only severely impair patients’ quality of life but also impose substantial economic burdens on health care systems [4]. Consequently, the identification of biomarkers that are capable of early risk stratification and disease progression prediction is essential for developing effective preventive and therapeutic strategies.

As a quantitative measure of HbA1c variability, the HGI may reflect the stability and fluctuations of glycemic control, thereby demonstrating correlations with CMDs risk. An increasing amount of evidence suggests a significant association between the HGI and CMDs [5, 6, 7], which may provide new research directions and potential intervention targets. This review synthesizes recent advances in the understanding of the role of the HGI in CMDs risk assessment, prognosis, and clinical management. By elucidating the mechanistic links between the HGI and CMDs, we aim to provide clinicians and researchers with a comprehensive perspective, thereby ultimately contributing to improve prevention and treatment of these debilitating diseases.

## 2 HGI

HbA1c, which is formed via nonenzymatic reactions between elevated blood glucose and hemoglobin, has been established as a primary biomarker for the monitoring of DM [8]. However, significant variability in the relationship between HbA1c and fasting plasma glucose (FPG) has been observed due to interindividual differences in glucose metabolism. To quantify this biological variation, Hempe et al. [9] introduced the HGI in 2002, which is defined as follows: HGI = measured HbA1c − predicted HbA1c [10]. The predictive model was derived from a previously validated linear regression equation (HbA1c = 0.435 × FPG (mmol/L) + 4.023; *r* = 0.699, *p* < 0.001) [11], which estimates expected HbA1c levels based on individual FPG measurements.

This methodological innovation offers two critical advantages: first, it statistically identifies individuals with HbA1c values that significantly deviate from FPG‐predicted levels; and second, it mitigates potential clinical misinterpretations arising from a sole reliance on HbA1c measurements [12]. Such discrepancies primarily stem from biological variables that affect HbA1c, including erythrocyte lifespan and glycation rates. The HGI framework introduces a crucial correction factor for precision DM management, thus enabling clinical assessments to better account for the fundamental biological determinants of glucose metabolism.

## 3 CMDs

CMDs represent a heterogeneous group of clinical syndromes arising from the interplay of genetic predispositions, environmental influences, behavioral factors, and metabolic dysregulation [13, 14]. Genetic determinants, including specific polymorphisms and familial predispositions, establish the biological foundation for CMDs development. Additionally, environmental triggers (such as pollution and dietary pattern shifts) further modulate disease susceptibility [15, 16]. Concurrently, modifiable behavioral risk factors (such as physical inactivity, poor nutrition, excessive alcohol consumption, and tobacco use) can significantly amplify CMDs progression [17, 18, 19].

At the metabolic level, dyslipidemia and glycemic instability emerge as pivotal pathogenic drivers of CMDs [20, 21, 22, 23]. Elevated lipid profiles promote atherogenesis, thereby increasing risks for CAD and stroke, whereas chronic hyperglycemia directly mediates diabetic pathogenesis and its complications. These elements interact via complex pathophysiological networks, thus collectively shaping the multifactorial etiology of CMDs.

CMDs encompass hypertension, DM, CAD, and related disorders [24]. These conditions not only share common mechanistic pathways but also exhibit strong clinical interdependencies, which often create vicious cycles of metabolic and cardiovascular deterioration [25]. For example, hypertensive patients frequently exhibit concurrent dyslipidemia and impaired glucose metabolism, which synergistically exacerbate end‐organ damage [26, 27].

**FIGURE 1:**
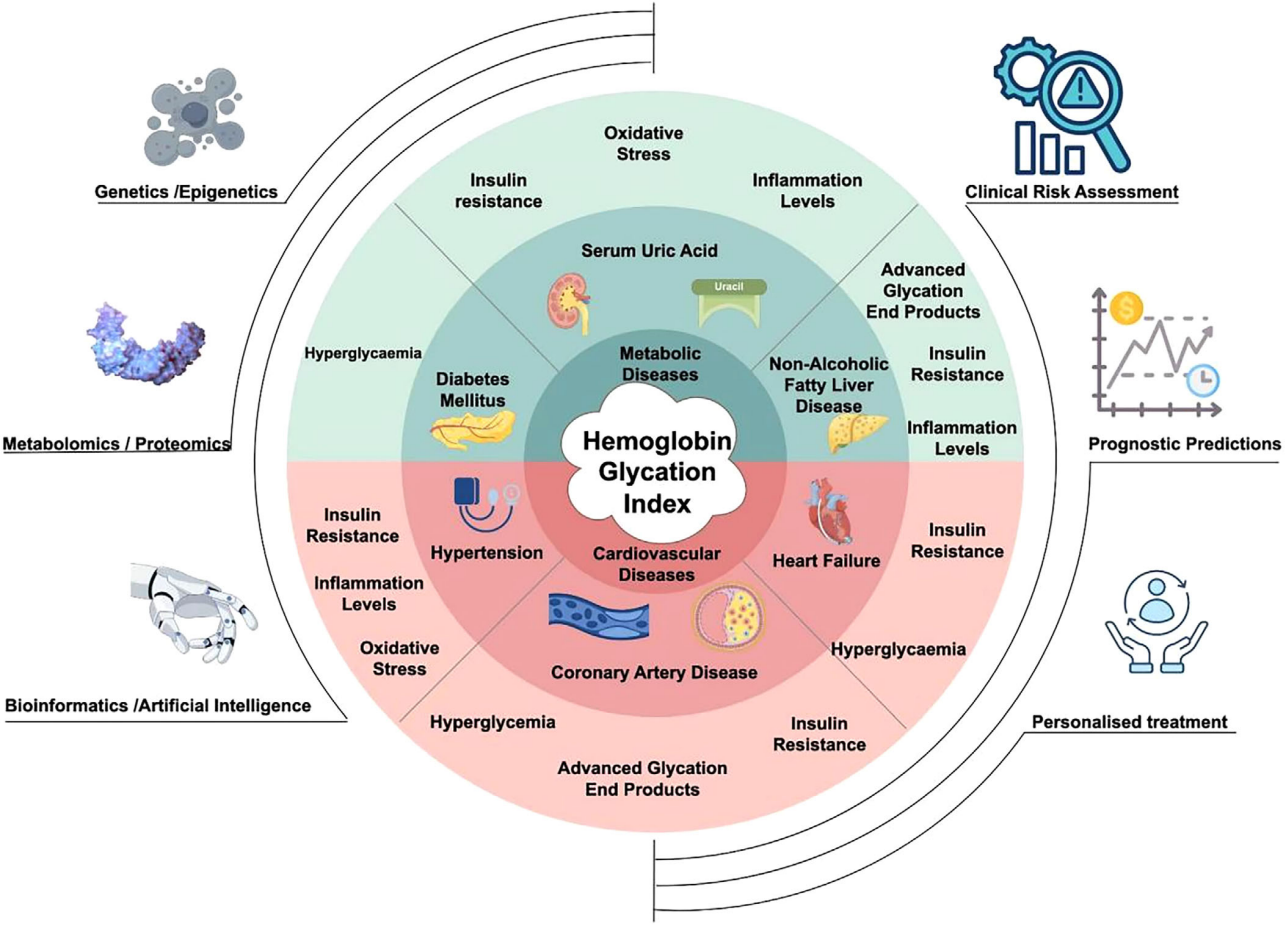
Association between the HGI and CMDs. This figure illustrates the relationship between the HGI and CMDs, the conditions and features comprising CMDs, and the possible pathophysiological mechanisms by which the HGI acts on CMDs. From inner to outer rings, the first pie chart delineates the bipartite classification of CMDs into CVDs and MDs. The upper sector of the second pie chart specifies MDs as encompassing DM, SUA, and NAFLD, while its lower sector categorizes CVDs as comprising hypertension, CAD, and HF. The third pie chart elucidates the mechanistic interplay between HGI and individual CMDs components. The left side of this figure extends the multidisciplinary cross‐application of the HGI (from top to bottom: genetics and epigenetics; metabolomics and proteomics; and bioinformatics and artificial intelligence), and the right side of the figure extends the clinical application of the HGI (from top to bottom: clinical risk assessment, prognostic predictions, and personalized treatment). CAD, coronary artery disease; CMD, cardiometabolic disease; CVD, cardiovascular disease; DM, diabetes mellitus; HF, heart failure; HGI, hemoglobin glycation index; MD, metabolic disorder; NAFLD, nonalcoholic fatty liver disease.

The persistently high global burden of CMDs morbidity and mortality necessitates the urgent identification of modifiable risk factors and the implementation of targeted interventions. Current strategies emphasize multimodal approaches combining lifestyle modifications (such as dietary optimization and structured exercise), pharmacological management, and personalized risk stratification. Such integrated interventions demonstrate significant potential for mitigating disease progression, improving clinical outcomes, and enhancing quality of life in at‐risk populations (Figure 1).

## 4 HGI and CVDs

### 4.1 HGI and CAD

CAD continues to be a predominant cause of mortality worldwide [28]. Although percutaneous coronary intervention and coronary artery bypass grafting can improve quality of life in affected individuals, individuals diagnosed with CAD still experience considerable remaining risks for future cardiovascular events [29]. This scenario underscores the critical need for reliable predictors of adverse clinical outcomes in this population. Accumulating evidence implicates glycemic variability in the pathogenesis and progression of CAD. Although fasting glucose and HbA1c constitute standard clinical measures for diabetic assessments, the variability in the diagnostic precision of these parameters leads to limitations regarding the use of these measures. To address this constraint, investigators have developed the HGI, which is an emerging biomarker demonstrating robust associations with CVDs risk and prognostic outcomes [30]. An investigation by Wen et al. [31] examined the prognostic relationship between the HGI and clinical outcomes in 10 598 patients with CAD. Participants were stratified into tertiles based on HGI values (low, intermediate, and high HGI groups), with subsequent evaluations of all‐cause mortality (ACM), cardiac mortality (CM), major adverse cardiac events (MACEs), and major adverse cardio‐cerebrovascular events being performed. Comparative analysis revealed significantly elevated risks of ACM (HR = 1.683, 95% CI: 1.179–2.404, *p* = 0.004) and CM (HR = 1.604, 95% CI: 1.064–2.417, *p* = 0.024) in the low‐HGI group compared with the intermediate‐HGI reference group. Conversely, the high‐HGI group demonstrated increased MACEs incidence (HR = 1.247, 95% CI: 1.023–1.521, *p* = 0.029) compared with the intermediate‐HGI group. These observations resulted in the establishment of a U‐shaped association between HGI levels and adverse outcomes encompassing ACM, CM, and MACEs, thereby substantiating the HGI as an independent predictor of MACEs risk in CAD patients. Furthermore, the results demonstrated that both low and high HGI levels are independently associated with adverse clinical outcomes in patients with critical CAD. These findings are consistent with the results reported by Lin et al. [32], demonstrating a U‐shaped association between HGI levels and MACEs incidence (nonlinear *p* = 0.014). Notably, patients with low HGI levels showed a 1.70‐fold higher risk of cardiovascular mortality (*p* < 0.05) (Table 1).

**TABLE 1:** Summary of previous studies on HGI and CAD.

Author (year)	Type of research	Sample size	Key findings	Conclusions
Cheng (2023) [11]	Retrospective cohort study	*n* = 1780	Versus moderate HGI, both low‐HGI (HR = 4.979, 95% CI: 1.865 ‐ 13.297, *p* = 0.001) and high‐HGI (HR = 2.918, 95% CI: 1.075 ‐ 7.922, *p* = 0.036) groups had elevated ACM risk.	(1) HGI predicts long‐term mortality in post‐PCI CAD patients. (2) It may optimize CAD clinical management.
Wen (2024) [31]	Prospective cohort study	*n* = 10 598	(1) Low HGI (HGI < −0.506) increased ACM (HR = 1.683, 95% CI: 1.179–2.404) and CM (HR = 1.604, 95% CI: 1.064–2.417). (2) High HGI (≥ 0.179) raised MACEs risk (HR = 1.247, 95% CI: 1.023–1.521).	HGI independently predicts mortality and MACEs in CAD patients.
Lin (2024) [32]	Prospective cohort study	*n* = 11 921	(1) Q2* (−0.840 ≤ HGI < −0.322) had lowest MACEs risk (*p* = 0.006). (2) Q1* (HGI < −0.840) and Q4*/Q5* (0.075 ≤ HGI < 0.790; HGI ≥ 0.790) showed higher MACEs risk (all *p* < 0.05). (3) Q1* exhibited 1.70‐fold increased ACM and CV death (both *p* < 0.05).	HGI levels exhibited a U‐shaped association with 3‐year MACEs incidence.
Wei (2024) [33]	Retrospective cohort study	*n* = 5260	(1) The Q2* group (−0.77 < HGI ≤ −0.37) had the lowest mortality. (2) Versus Q2*: Q1* (HGI < −0.77) showed higher 365‐day (HR = 1.48, 95% CI: 1.19–1.85) and 30‐day mortality (HR = 1.96, 95% CI: 1.38–2.78; both *p* < 0.001) Q4* (HGI > 0.25) had increased 365‐day mortality (HR = 1.31, 95% CI: 1.02–1.69, *p* < 0.05).	(1) Both high and low HGI impaired survival in CAD patients, particularly at low levels. (2) HGI and mortality risk showed U‐shaped association in critical CAD.

*Note*: Q1–Q4/Q5, participants were stratified into quartiles (Q1–Q4) or quintiles (Q1–Q5) based on their HGI values.

Abbreviations: ACM, all‐cause mortality; CAD, coronary artery disease; CM, cardiac mortality; CV, cardiovascular; HGI, hemoglobin glycation index; MACE, major adverse cardiac event.

#### 4.1.1 Mechanistic Links Between HGI and CAD

**FIGURE 2:**
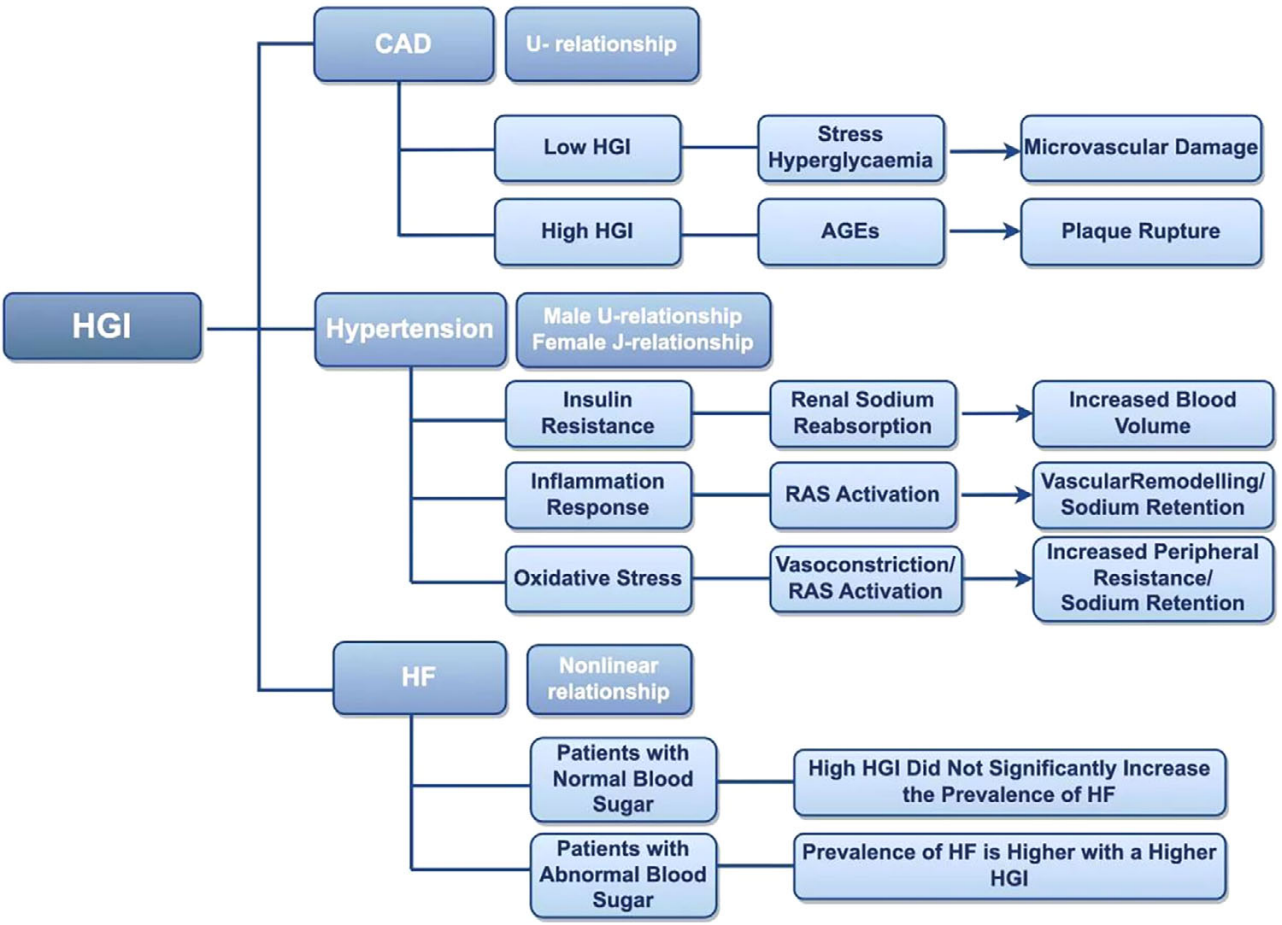
The association between the HGI and CVDs incidence. This figure summarizes the relationships between the HGI and CAD, hypertension, and HF, along with their underlying mechanisms. For CAD, the HGI demonstrates a U‐shaped association, wherein both excessively high and low HGI levels may increase the risk of coronary mortality and MACEs. The mechanisms underlying poor prognosis at low and high HGI levels are separately delineated. With respect to hypertension, the association between the HGI and outcomes is sex dependent; specifically, a U‐shaped relationship is observed in males (with elevated mortality risk being noted at both extremes of the HGI), whereas a J‐shaped relationship exists in females (where moderate HGI levels are correlated with reduced cardiovascular and mortality risks). Potential pathophysiological mechanisms are also discussed. For HF, elevated HGI levels did not significantly increase the incidence of HF in normoglycemic individuals. However, in patients with dysglycemia, higher HGI levels are associated with greater HF incidence. AGE, advanced glycation end product; CAD, coronary artery disease; CVD, cardiovascular disease; HF, heart failure; HGI, hemoglobin glycation index; MACE, major adverse cardiac event; RAS, renin–angiotensin system.

Regarding the mechanistic basis for the influence of the HGI on CAD prognosis, studies have suggested that acute stress responses in critically ill patients induce rapid glucose elevation, with this stress‐induced hyperglycemia exacerbating coronary disease severity via multiple pathways, including the induction of endothelial dysfunction, aggravation of microvascular obstruction, and direct vascular endothelial injury, all of which demonstrate independent associations with short‐term adverse clinical outcomes [34, 35]. A study by Wei et al. [33] further elucidated the mechanistic link between low HGI and increased short‐term mortality in critically ill CAD patients, which is potentially mediated via stress‐induced hyperglycemia. Conversely, the association between elevated HGI levels and increased MACEs risk in CAD patients may be attributable to the pathological effects of advanced glycation end products (AGEs) [36]. AGEs promote cardiovascular pathogenesis through multiple pathways, including disturbances of vascular compliance and myocardial elasticity; the impairment of vasodilation via reduced nitric oxide production; the enhancement of foam cell formation via decreased low‐density lipoprotein clearance; and the exacerbation of endothelial dysfunction via oxidative stress (OS) and inflammatory cascades [37]. Thus, elevated HGI levels may reflect a metabolically unstable state characterized by insulin resistance (IR), glycemic variability, disrupted energy metabolism, and OS, with the latter effect representing a well‐established cardiovascular risk factor [38]. Despite the existing mechanistic hypotheses proposed by various investigators regarding the role of the HGI in CAD prognosis, the precise underlying mechanisms remain incompletely understood. Consequently, future research should prioritize comprehensive investigations into the specific pathways through which the HGI influences CAD outcomes. Such mechanistic insights could facilitate the development of more effective preventive and therapeutic strategies, thereby ultimately leading to substantial reductions in both mortality rates and adverse vascular events among CAD patients (Figure 2).

### 4.2 HGI and Hypertension

Hypertension has emerged as a critical global health concern, and it currently affects over 1.3 billion individuals worldwide. As a predominant risk factor for CVDs, it significantly contributes to increase global mortality rates [39]. Lifestyle modifications serve as the cornerstone for both the prevention and management of elevated blood pressure [40, 41]. These interventions not only demonstrate efficacy in reducing blood pressure and optimizing hypertension control but also confer substantial benefits for overall cardiovascular health. Consequently, major hypertension management guidelines universally recommend lifestyle modifications as first‐line strategies for cardiovascular risk reduction [42]. Hypertensive patients frequently exhibit concurrent glucose metabolism abnormalities [43], thus indicating that glycemic control is a crucial component of comprehensive health management. As a novel quantitative marker of glycemic control, the HGI is significantly associated with hypertension. Clinical investigations have further substantiated the association between the HGI and hypertension. For example, the study by Jin et al. [44] enrolled 3155 patients with stable CAD and stratified them into six groups based on HGI tertiles and hypertension statuses for MACEs follow‐up. Among the 8150 person‐years of follow‐up, 328 cardiovascular events were recorded. Although no significant difference in MACEs incidence (HR = 1.195, 95% CI: 0.885–1.613, *p* > 0.05) was observed between the low‐ and intermediate‐HGI groups, the high‐HGI group demonstrated elevated MACEs risk (HR = 1.354, 95% CI: 1.010–1.814, *p* < 0.05). When participants were categorized by combined parameter status, both the intermediate HGI with hypertension subgroup and the high HGI with hypertension subgroup exhibited significantly increased cardiovascular risks (HR = 2.558, 95% CI: 1.469–4.457; HR = 2.241, 95% CI: 1.285–43.908; all *p* < 0.05) compared with the reference group (low HGI with normotension). These findings suggest that coexisting hypertension may modify the predictive value of the HGI for cardiovascular outcomes in stable CAD patients with DM and prediabetes. Additional investigations have examined the association between the HGI and mortality risk in hypertensive patients, with a particular emphasis on sex‐specific effects. Compared with the lowest HGI quintile (Q0), the highest quintile (Q4) exhibited significantly elevated cardiovascular mortality (HR = 1.34, 95% CI: 1.01–1.76). Notably, the Q2 (HR = 0.79, 95% CI: 0.69–0.92) and Q3 groups (HR = 0.86, 95% CI: 0.74–0.99) demonstrated reduced ACM rates. These findings have important clinical implications for hypertension management strategies. Analysis by sex revealed a U‐shaped relationship (HGI < −0.271: Lower CVDs [HR = 0.64, 95% CI: 0.44–0.93] and ACM [HR = 0.84, 95% CI: 0.71–0.99]; HGI ≥ 0.115: Higher CVDs [HR = 1.48, 95% CI: 1.23–1.79] and ACM [HR = 1.41, 95% CI: 1.24–1.60]) between the HGI and mortality risk in male populations, wherein both elevated and diminished HGI levels were associated with increased mortality. In contrast, female subjects demonstrated an inverse correlation between moderate HGI levels and reduced cardiovascular risk. This phenomenon is potentially attributable to estrogen‐mediated cardioprotective effects. Notably, hormonal variations appear to attenuate the mortality risk associated with elevated HGI levels in female populations [45]. The observed sexual dimorphism may further stem from fundamental differences in adipose tissue distribution and energy metabolism between the sexes. Specifically, males typically exhibit a predominance of visceral fat, whereas females generally demonstrate greater subcutaneous adipose accumulation [46]. This anatomical distinction has clinical importance, as visceral adiposity is mechanistically linked to IR, chronic inflammation, and consequent cardiovascular pathogenesis [47, 48, 49]. Research has demonstrated that an elevated visceral adipose tissue (VAT) to subcutaneous adipose tissue (SAT) ratio is associated with the development of CVDs [49]. However, given the paucity of investigations examining these sex‐specific differences, further research is warranted to elucidate their implications for cardiovascular health. Future studies may enable refined hypertension risk stratification via comprehensive biomarker assessments, thus ultimately facilitating the development of personalized prevention and management strategies for this vulnerable demographic (Table 2).

**TABLE 2:** Summary of previous studies on HGI and hypertension.

Author (year)	Type of research	Sample size	Key findings	Conclusions
Shangguan (2024) [45]	Retrospective cohort study	*n* = 7607	(1) HGI showed a U‐shaped relationship with CVDs and ACM after adjustment. (2) Sex‐specific analyses revealed: ① Males: a. CVDs mortality and ACM: U‐shaped relationship b. HGI < −0.271: Lower CVDs (HR = 0.64, 95% CI: 0.44–0.93) and ACM (HR = 0.84, 95% CI: 0.71–0.99) c. HGI ≥ 0.115: Higher CVDs (HR = 1.48, 95% CI: 1.23–1.79) and ACM (HR = 1.41, 95% CI: 1.24–1.60) ②Females: a. CVDs mortality: J‐shaped relationship b. ACM: L‐shaped relationship c. HGI < threshold: Reduction in ACM risk (HR = 0.66, 95% CI: 0.56–0.77) d. HGI ≥ threshold: Elevated CVDs mortality risk (HR = 1.39, 95% CI: 1.12–1.72).	Optimal HGI levels are clinically important in hypertension, serving as a validated biomarker for CV mortality and ACM.
Mi (2020) [50]	Cross‐sectional study	*n* = 1777	(1) Q4* vs. Q1*: elevated risk of incident hypertension (aOR = 1.87, 95% CI: 1.26–2.78). (2) Per 1‐unit HGI increase: 1.16‐fold risk of acquiring hypertension (OR = 1.16, 95% CI: 1.06–1.27).	High HGI independently associates with hypertension risk.
Jin (2019) [51]	Prospective cohort study	*n* = 8150	Compared to the low HGI with normotensive group: (1) Moderate HGI + hypertension showed elevated CVDs risk (HR = 2.558, 95% CI: 1.469–4.457, *p* < 0.05). (2) High HGI + hypertension showed elevated CVDs risk (HR = 2.241, 95% CI: 1.285–3.908, *p* < 0.05).	Hypertension may modify HGI's predictive value for stable CAD in DM/prediabetes.

*Note*: Q1–Q4, participants were stratified into quartiles (Q1–Q4) based on their HGI values.

Abbreviations: ACM, all‐cause mortality; CAD, coronary artery disease; CVD, cardiovascular disease; DM, diabetes mellitus; HGI, hemoglobin glycation index.

#### 4.2.1 Mechanistic Links Between HGI and Hypertension

This relationship may originate from multiple pathophysiological mechanisms, including IR, chronic inflammation, and OS [52, 53, 54, 55]. These mechanisms play pivotal roles in the pathogenesis of hypertension.

First, IR influences blood pressure regulation via several distinct pathways: augmentation of sympathetic nervous system activity through adrenergic system stimulation; suppression of prostacyclin generation in adipose tissue, thereby attenuating its vasodilatory effects; and modulation of Na^+^/K^+^‐ATPase activity that enhances vascular smooth muscle cell sensitivity to vasoactive substances (e.g., catecholamines and angiotensin II), collectively culminating in increased peripheral vascular resistance [56]. Second, OS plays a pivotal role in hypertensive pathogenesis. The disequilibrium between reactive oxygen species (ROS) production and antioxidant defense mechanisms leads to: (1) endothelial dysfunction; (2) proinflammatory state induction; (3) enhanced vasoconstriction; and (4) cardiovascular tissue remodeling. These pathological alterations collectively contribute to elevated blood pressure and target organ damage [57]. Furthermore, chronic inflammatory responses participate in hypertension pathophysiology through elevated inflammatory markers (including CRP, TNF‐α, IL‐6, and IL‐1β), with principal mechanisms comprising: impairment of endothelium‐dependent vasodilation; promotion of vascular smooth muscle cell proliferation; and increased arterial stiffness [58].

Critically, these mechanisms do not operate in isolation but rather exhibit extensive crosstalk and mutual reinforcement. The triad of IR, OS, and chronic inflammation converges to induce endothelial dysfunction, accelerate atherogenesis, and ultimately elevate the risk of hypertension and its cardiovascular complications.

### 4.3 HGI and HF

Heart failure (HF) represents a complex clinical syndrome characterized by impaired cardiac pumping capacity, thereby resulting in systemic circulatory dysfunction [59, 61]. Emerging evidence from recent mechanistic studies underscores the significant association between glycemic control and HF pathogenesis [59]. In this context, the HGI (as a biomarker reflecting long‐term glycemic regulation) has garnered increasing attention for its potential role in HF development. Multiple clinical investigations have established both hyperglycemia and IR as independent risk factors for HF onset and progression [62, 63]. Patients with DM have a significantly greater risk of developing HF compared to nondiabetic individuals. As an indicator of glycemic control, the HGI is strongly associated with HF incidence. However, the current research findings regarding the relationship between the HGI and HF prognosis are inconsistent. For example, an investigation of patients with acute decompensated HF revealed that elevated HGI levels were associated with reduced ACM (HR = 0.720, 95% CI: 0.563–0.921, *p* = 0.009) and cardiovascular mortality (HR = 0.619, 95% CI: 0.445–0.861, *p* = 0.004). This observation may be attributed to the fact that the majority of the participants in this study demonstrated HbA1c levels below 7.0% [64]. In contrast, other studies have revealed differential predictive effects of the HGI on HF across varying glucose metabolic states. Specifically, elevated HGI levels did not significantly increase the incidence of HF in individuals with normoglycemia. Conversely, higher HGI levels were associated with greater HF incidence in both prediabetic (OR = 1.59, 95% CI: 1.15–2.20) and diabetic populations (OR = 1.61, 95% CI: 1.14–2.28) [65]. These discrepancies may be due to interstudy variations in sample sizes, baseline comorbidities, and treatment regimens. Nevertheless, the maintenance of optimal glycemic control remains clinically important for improving HF patient outcomes. Given the established association between the HGI and HF, clinicians should incorporate glycemic monitoring into HF management strategies. Moreover, regular assessments of HGI levels will enable the evaluation of the efficacy of glycemic control, thereby facilitating timely therapeutic adjustments to mitigate both HF incidence and disease severity. Furthermore, optimized glycemic control in established HF patients may yield additional prognostic benefits. As a novel metric capturing interindividual variations in glycemic control, the HGI demonstrates potential clinical utility for both risk stratification and therapeutic monitoring in HF patients (Table 3).

**TABLE 3:** Summary of previous studies on HGI and HF.

Author (year)	Type of research	Sample size	Key findings	Conclusions
Wang (2024) [65]	Retrospective cohort study	*n* = 9847	Elevated HGI increased HF risk in: (1) General population (OR = 1.33, 95% CI: 1.08–1.63), (2) Prediabetes (OR = 1.59, 95% CI: 1.15–2.20), (3) DM (OR = 1.61, 95% CI: 1.14–2.28), (4) Elevated HGI showed no significant HF risk in normoglycemia.	(1) Elevated HGI increases HF prevalence in hypertension. (2) This association is restricted to prediabetic/diabetic individuals, absent in normoglycemic subjects.
Guo (2025) [66]	Retrospective cohort study	*n* = 2846	(1) Elevated HGI was significantly associated with higher 30‐day (aHR = 2.36, 95% CI: 1.74–3.20, *p* < 0.001) and 365‐day mortality (aHR = 1.40, 95% CI: 1.16–1.68, *p* < 0.001). (2) Per 1‐unit increment in HGI was associated with a 1.42‐fold increased risk of 30‐day mortality (aHR = 1.42, 95% CI: 1.28–1.57, *p* < 0.001) and a 1.19‐fold increased risk of 365‐day mortality (aHR = 1.19, 95% CI: 1.11–1.28, *p* < 0.001).	Elevated HGI independently predicted higher 30‐ and 365‐day mortality, with levels > 0.709 identifying high‐risk patients.
Wang (2025) [67]	Retrospective cohort study	*n* = 10 889	HGI and CHF risk showed a U‐shaped relationship (*p* nonlinear = 0.0001), with increased risk per unit HGI above −0.140 (OR = 1.39, 95% CI: 1.22–1.58).	HGI shows a U‐shaped relationship with CHF risk (threshold: −0.140) in DM/prediabetes, suggesting its potential as an early cardiovascular risk biomarker.
Wang (2025) [68]	Retrospective cohort study	*n* = 8098	(1) Per‐unit HGI increase showed 12% lower in‐hospital mortality (OR = 0.88, 95% CI: 0.83–0.93) and 3% reduced 1‐year ACM (HR = 0.97, 95% CI: 0.94–1.00). (2) RCS analysis showed a J‐shaped relationship between the HGI and mortality, with increased risk at very low HGI levels.	(1) HGI demonstrated a robust association with ACM (*p* < 0.001). (2) RCS analysis revealed a J‐shaped relationship between the HGI and mortality. (3) Patients with extremely low HGI levels demonstrated the highest mortality risk. (4) HGI provides incremental prognostic value beyond conventional markers.
Sun (2025) [69]	Retrospective cohort study	*n* = 985	(1) HGI showed significant inverse associations with mortality at: ①30 days (HR = 0.79, 95% CI: 0.67–0.92, *p* = 0.003) ②60 days (HR = 0.83, 95% CI: 0.72–0.96, *p* = 0.011) ③90 days (HR = 0.86, 95% CI: 0.75–0.99, *p* = 0.031) ④365 days (HR = 0.97, 95% CI: 0.86–1.09, *p* = 0.611). (2) RCS analysis revealed a U‐shaped relationship between the HGI and clinical outcome events.	In CHF, elevated HGI showed greater cardioprotection versus lower levels, correlating with decreased short‐term mortality.

Abbreviations: CHF, congestive heart failure; HF, heart failure; HGI, hemoglobin glycation index.

#### 4.3.1 Mechanistic Links Between HGI and HF

The association between the HGI and HF is primarily mediated through the following mechanisms: Elevated HGI levels reflect poor glycemic control and an increased propensity for nonenzymatic protein glycation, leading to pathological accumulation of AGEs. Through binding with the receptor for AGEs (RAGE), these compounds activate the NADPH oxidase system, resulting in excessive ROS production, while simultaneously triggering the NF‐κB signaling pathway to promote the release of proinflammatory cytokines (TNF‐α, IL‐6) and activating Toll‐like receptor 4‐mediated inflammatory pathways. This creates a vicious cycle of OS and chronic inflammation. These pathological processes induce multifaceted damage: on the one hand, they impair endothelial function by reducing nitric oxide bioavailability and increasing vascular stiffness; on the other hand, they exacerbate metabolic dysregulation by inhibiting mitochondrial respiration and promoting oxidative modification of low‐density lipoproteins. The persistent oxidative damage and inflammatory state ultimately drive myocardial fibrosis and ventricular remodeling, leading to progressive deterioration of cardiac structure and function, thereby increasing the risk of HF development [65, 66].

## 5 HGI and Metabolic Disorders (MDs)

### 5.1 HGI and DM

**FIGURE 3:**
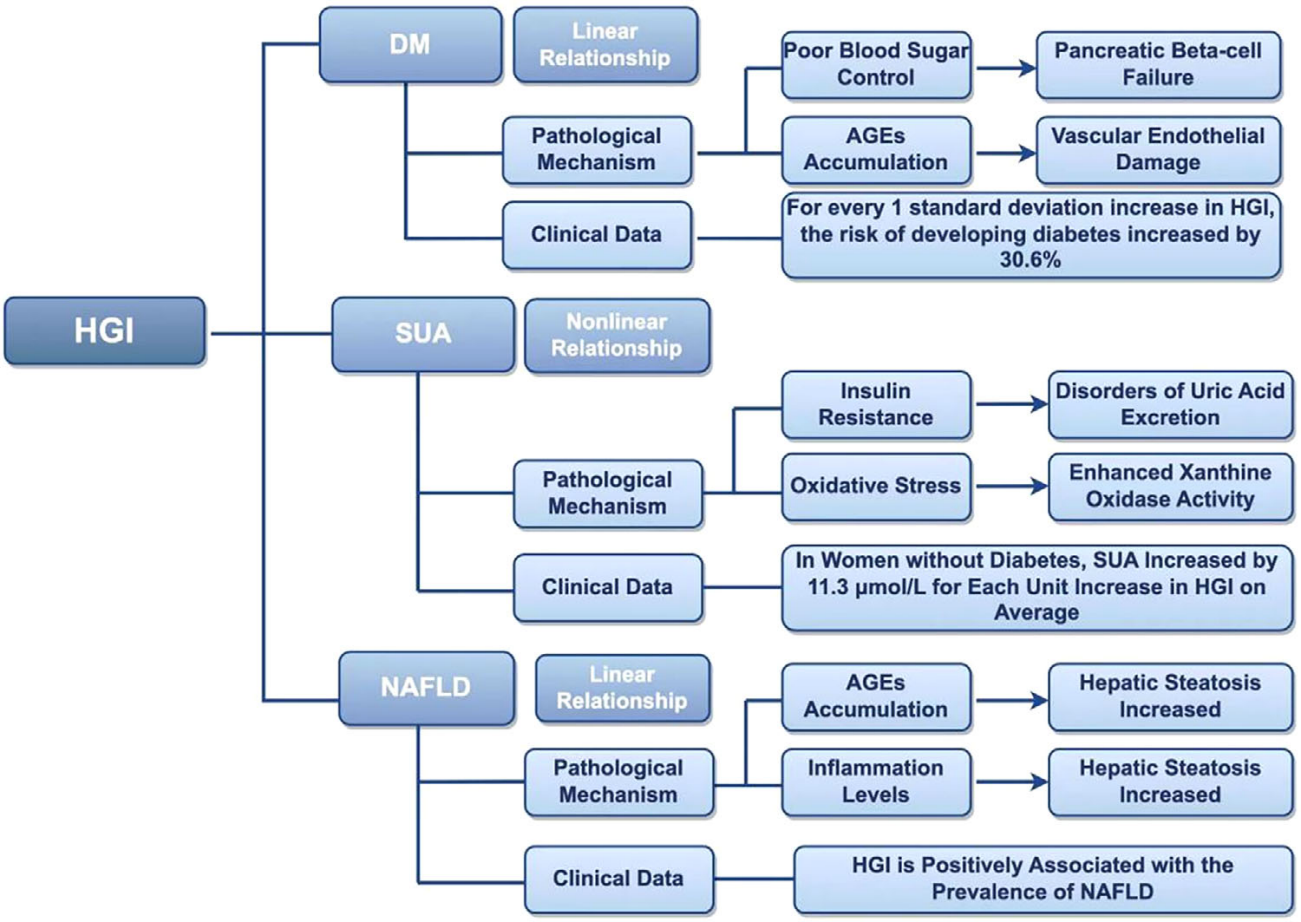
The association between the HGI and MDs. The figure delineates the relationships between HGI and DM, SUA, and NAFLD, including their underlying mechanisms and supporting clinical evidence. For DM and NAFLD, HGI exhibits a linear association, whereas a nonlinear relationship is observed for SUA. Critically, elevated HGI levels consistently correlate with increased mortality risks and adverse clinical outcomes across all three metabolic disorders. The figure further elaborates the pathophysiological mechanisms involved and substantiates these associations with clinical data. AGE, advanced glycation end product; DM, diabetes mellitus; HGI, hemoglobin glycation index; MD, metabolic disorder; NAFLD, nonalcoholic fatty liver disease; SUA, serum uric acid.

The HGI is quantified as the difference between measured HbA1c levels and predicted HbA1c values derived from FPG, thereby reflecting both the magnitude and duration of glycemic fluctuations (Figure 3). This parameter serves as a clinically significant indicator of long‐term glycemic control. DM, which is characterized by chronic hyperglycemia, demonstrates a strong pathophysiological association with the HGI [70]. The fundamental mechanism of DM involves the dysregulation of glucose metabolism caused by IR and impaired insulin secretion [71]. As a sensitive marker of glycemic variability, elevated HGI levels indicate greater glucose fluctuations and may predict an increased risk of DM. A longitudinal study of 7345 nondiabetic participants aged > 40 years with a median follow‐up of 3.24 years revealed a significant association between elevated HGI levels and increased incidence of DM. Specifically, each standard deviation increase in the HGI level corresponded to a 30.6% greater risk of DM development (HR = 1.306, 95% CI: 1.232–1.384). These findings suggest the potential utility of the HGI as a biomarker for identifying high‐risk individuals and imply its pathophysiological relevance to DM pathogenesis [72]. Additional studies have demonstrated that elevated HGI levels in diabetic patients are correlated with greater glycemic variability and more severe disease progression. A higher HGI level is associated with both an increased risk of hypoglycemic events in diabetic patients and elevated susceptibility to chronic vascular complications (regardless of DM status) [73]. The monitoring of the HGI enables clinicians to obtain a comprehensive assessment of the glycemic control statuses of patients, thereby facilitating the development of personalized treatment strategies. Furthermore, the HGI serves as a valuable reference parameter for evaluating therapeutic efficacy in DM management, thus allowing for timely treatment optimization to enhance clinical outcomes. Additionally, the HGI demonstrates predictive utility for DM‐related complications, with elevated HGI levels being associated with higher incidence rates of diabetic complications [72, 74]. CVDs represent one of the most prevalent complications in patients with DM, with elevated HGI levels being correlated with significantly increased cardiovascular risk [75]. Diabetic retinopathy (a common diabetic ocular complication) demonstrates an increased incidence in patients with higher HGI values, with this complication frequently progressing to retinal vasculopathy, which may result in visual impairment or blindness [76]. Similarly, renal complications in diabetic patients are strongly associated with HGI levels, whereas chronic kidney disease patients with elevated HGI levels exhibit accelerated deterioration of renal function and a greater propensity for uremia development [77].

#### 5.1.1 Mechanistic Links Between HGI and DM

The association between the HGI and DM can be elucidated through multiple mechanistic dimensions. At the pathophysiological level, HGI quantifies the discrepancy between measured HbA1c levels and predicted values, reflecting an individual's propensity for glycation. This essentially uncovers three fundamental characteristics: impaired glucose metabolic capacity; altered β‐cell function; and heightened nonenzymatic glycation activity. Molecular investigations reveal that aberrant HGI levels contribute to disease progression through two pivotal pathways: the “metabolic memory” effect, characterized by sustained activation of protein glycation cascades that promote accumulation of AGEs and initiate a vicious cycle of OS and inflammation; and direct islet dysfunction, manifested through exacerbated β‐cell dedifferentiation, suppressed proinsulin biosynthesis, and accelerated islet amyloid polypeptide deposition [78, 79].

### 5.2 HGI and SUA

Serum uric acid (SUA) is an MD characterized by dysregulated purine metabolism, thereby leading to elevated SUA concentrations. This condition is clinically diagnosed when SUA levels exceed the upper limits of the normal range (> 420 µmol/L in males or > 357 µmol/L in females) [80]. Elevated SUA levels are clinically significant not only for precipitating gout (with manifestations of joint erythema, swelling, warmth, and pain) but also for inducing renal impairment and vascular endothelial dysfunction, as well as for exacerbating both microvascular and macrovascular complications in individuals with diabetes [81]. Research has demonstrated that the relationship between the HGI and SUA is influenced by sex and diabetes status. In women, the effect of the HGI on SUA levels varies depending on the presence of diabetes, whereas in men, this interaction does not reach statistical significance. Specifically, a positive correlation between the HGI and SUA levels was observed in nondiabetic women, with each 1‐unit increase in HGI corresponding to an 11.3 µmol/L elevation in SUA (*p* < 0.001), whereas the association exhibited a nonlinear pattern in women with DM (*p* < 0.002). In contrast, no significant relationship was observed in either diabetic (*p* < 0.11) or nondiabetic men (*p* < 0.94) [82]. In summary, both diabetic individuals and the general population should maintain glycemic control. The regulation of blood glucose levels may help to reduce the risk of SUA, particularly in nondiabetic women. Although further research is needed to fully elucidate the relationship between the HGI and SUA, current evidence suggests that for diabetic patients, regular monitoring of these two parameters and corresponding adjustments in therapeutic strategies demonstrate significant clinical value for preventing and managing complications.

#### 5.2.1 Mechanistic Links Between HGI and SUA

The mechanistic link between the HGI and hyperuricemia stems from shared pathophysiological underpinnings: Elevated SUA levels promote IR in multiple tissues (including pancreatic β‐cells, skeletal muscle, and adipose tissue) by inducing OS and stimulating proinflammatory cytokine production, while concurrently impairing intrarenal hemodynamics. HGI, as a marker of glycation processes, similarly correlates with OS and chronic inflammation. These factors establish a vicious cycle: SUA accelerates nonenzymatic protein glycation through inflammatory pathway activation and OS, thereby increasing HGI. Conversely, the accumulation of AGEs reflected by elevated HGI exacerbates IR and inflammatory states, which further suppress uric acid excretion and promote SUA elevation. This bidirectional interplay is particularly pronounced in renal tissue, where SUA‐induced hemodynamic abnormalities and HGI‐related metabolic memory effects synergistically compromise renal function, creating a self‐perpetuating state of impaired urate excretion that sustains both hyperuricemia and glucose MDs [83, 84, 85, 86, 87, 88].

### 5.3 HGI and NAFLD

Nonalcoholic fatty liver disease (NAFLD) is a pathological syndrome characterized by the excessive accumulation of fat in hepatocytes in the absence of excessive alcohol consumption or other well‐defined causes. Previous studies have demonstrated an association between NAFLD and MDs linked to IR, including type 2 DM, obesity, and metabolic syndrome [89]. Furthermore, NAFLD predisposes individuals to atherosclerosis and is associated with an elevated risk of CVDs [90]. Recent studies have demonstrated that elevated HGI levels may be associated with the development and progression of NAFLD. Some researchers stratified participants into quartiles based on their HGI levels to further investigate the association between HGI and NAFLD risk. The analysis revealed a significantly higher prevalence of NAFLD in the highest HGI quartile (OR = 1.564, 95% CI: 1.350–1.813, *p* < 0.001), suggesting that elevated HGI is independently associated with NAFLD [91]. In a large‐scale study encompassing 14 280 participants stratified into quartiles (Q1–Q4) based on ascending HGI values, significant associations with NAFLD were observed. Comparative analysis revealed that NAFLD patients exhibited markedly higher HGI levels than their non‐NAFLD counterparts. The prevalence of NAFLD demonstrated a progressive increase across HGI quartiles, with Q2, Q3, and Q4 all showing higher rates compared to the reference Q1 group (*p* < 0.001). Notably, the highest NAFLD prevalence occurred in the top HGI quartile (Q4), establishing a clear dose–response relationship between elevated HGI levels and increased NAFLD risk [92]. Previously, Hu et al. [93] developed a predictive model for NAFLD risk based on the HGI. Their findings demonstrated a significant association between HGI and NAFLD incidence, with elevated HGI levels showing a positive correlation with hepatic steatosis risk (OR = 1.172, 95% CI: 1.074–1.279). Building upon this association, the researchers constructed a comprehensive risk assessment algorithm: NAFLD risk score = 0.363 × HGI + 0.744 × lnTG + 1.674 × lnFPG + 0.213 × BMI + 1.361 × lnALT − 0.425 × HDL‐C + 0.130 × WBC − 14.381. These results position HGI as a promising biomarker for future NAFLD risk stratification in clinical practice. These findings have important implications for the management and treatment of NAFLD in diabetic patients. Notably, although the relationship between the HGI and NAFLD has been investigated (to some extent), current studies still demonstrate several limitations. For example, most of the studies were single‐center observational investigations with limited sample sizes; moreover, the diagnosis of NAFLD primarily relied on ultrasound imaging, with the variability in the accuracy of this technique potentially affecting the conclusions of these studies. Therefore, future research should incorporate larger cohorts, more precise diagnostic methods, and more in‐depth mechanistic studies to further validate and expand the clinical utility of the HGI in NAFLD risk assessments (Table 4).

**TABLE 4:** Summary of previous studies on HGI and NAFLD.

Author (year)	Type of research	Sample size	Key findings	Conclusions
Yoo (2019) [91]	Retrospective cohort study	*n* = 14 465	(1) NAFLD prevalence rose progressively with HGI quartiles (Q1*–Q4*: 24.8%, 29.7%, 32.6%, and 40.6% respectively; *p* < 0.001). (2) Participants in the highest HGI quartile exhibited a significantly increased prevalence of NAFLD (OR = 1.564, 95% CI: 1.350–1.813, *p* < 0.001).	Elevated HGI levels exhibit an independent association with NAFLD.
Xing (2023) [92]	Retrospective cohort study	*n* = 14 280	(1) HGI constitutes an independent risk factor for NAFLD (OR = 2.811, 95% CI: 2.313–3.417, *p* < 0.001). (2) NAFLD prevalence increases progressively with higher HGI levels (*p* < 0.001).	HGI serves as an independent risk factor for NAFLD.
Hu (2019) [93]	Cross‐sectional study	*n* = 3936	(1) Elevated HGI independently predicted hepatic steatosis risk (OR = 1.172, 95% CI: 1.074–1.279). (2) An HGI‐based risk score (0.363 × HGI + 0.744 × lnTG + 1.674 × lnFPG + 0.213 × BMI + 1.361 × lnALT − 0.425 × HDL‐C + 0.130 × WBC − 14.381) stratified NAFLD risk.	(1) NAFLD independently associates with HGI in Chinese nondiabetic adults. (2) The NAFLD risk score predicts disease risk in nondiabetic cohorts.
Fiorentino (2017) [94]	Retrospective cohort study	*n* = 1120	Subjects in the highest HGI quartile demonstrated a 1.6‐fold increased risk of hepatic steatosis (95% CI: 1.03–2.41, *p* = 0.03) compared to those in the lowest quartile.	Elevated HGI levels are associated with increased risk of hepatic steatosis in nondiabetic individuals.

*Note*: Q1–Q4, participants were stratified into quartiles (Q1–Q4) based on their HGI values.

Abbreviations: HGI, hemoglobin glycation index; NAFLD, nonalcoholic fatty liver disease.

#### 5.3.1 Mechanistic Links Between HGI and NAFLD

HGI likely contributes to the pathogenesis and progression of NAFLD through multiple interconnected mechanisms. (1) AGE‐mediated pathways: In diabetic patients, accumulated AGEs bind to RAGE, activating downstream signaling that promotes ROS generation and inflammatory responses. This process impairs insulin signaling pathways, thereby facilitating NAFLD development and progression [95]. (2) Chronic inflammation and IR: Chronic inflammation plays a pivotal role in NAFLD pathogenesis, with higher HGI values correlating with elevated inflammatory markers, suggesting its importance in HGI‐induced liver injury. The well‐established association between IR and NAFLD is further exacerbated by inflammatory responses that disrupt insulin signaling, promoting hepatic lipid accumulation while simultaneously inducing endoplasmic reticulum and OS—ultimately driving more severe liver pathology [94].

## 6 Summary and Perspectives

In summary, the HGI is closely associated with the onset and progression of CMDs and is a potential predictor of CMDs risk. Elevated HGI levels not only indicate suboptimal long‐term glycemic control but are also associated with increased risks of cardiovascular diseases (including hypertension, CAD, and HF) and metabolic diseases (such as DM and SUA). Consequently, the HGI has become an important parameter in both DM management and early CMDs screening and risk assessment. However, current research on the HGI is incomplete. To further elucidate the relationship between the HGI and CMDs, future investigations should prioritize large‐scale, multicenter clinical trials to evaluate the feasibility and efficacy of HGI modulation in preventing or delaying CMDs onset. These studies can provide critical evidence for the development of more precise therapeutic strategies. Moreover, significant knowledge gaps persist in elucidating the mechanistic relationships between the HGI and CMDs. Future investigations should employ an integrated research framework addressing fundamental biological mechanisms, genetic and environmental interactions, clinical intervention strategies, and the development of novel biomarker panels to advance this field.

## Author Contributions

Q.‐Y.W. searched the literature and drafted the manuscript. Q.S. and Q.W. conceived and designed the review. J.N. and W.‐Z.H. constructed the figures. Q.S., Q.W., and L.‐R.M. made critical revisions of the review. All the authors contributed to the article and approved the final version for submission.

## Ethics Statement

The authors have nothing to report.

## Conflicts of Interest

The authors declare no conflicts of interest.

## Data Availability

The authors have nothing to report.
